# Crucial role of treatment with palliative intent for a patient with advanced thymic carcinoma

**DOI:** 10.3892/ol.2014.2217

**Published:** 2014-06-03

**Authors:** MAKOTO NAGAMATA, YUSUKE OKUMA, YUKO YAMADA, YUKIO HOSOMI, TSUNEKAZU HISHIMA

**Affiliations:** 1 Department of Thoracic Oncology and Respiratory Medicine, Tokyo Metropolitan Cancer and Infectious Diseases Center Komagome Hospital, Tokyo, Japan; 2 Department of Pathology, Tokyo Metropolitan Cancer and Infectious Diseases Center Komagome Hospital, Tokyo, Japan

**Keywords:** thymic carcinoma, chemotherapy, radiotherapy, whole-brain radiotherapy

## Abstract

Thymic carcinoma is a rare cancer that is more aggressive and shows a poorer prognosis compared with thymoma. Molecular analysis has demonstrated that this entity is clearly distinct from thymoma. However, no definitive clinical management has been reported, and the roles of chemotherapy and radiotherapy for advanced thymic carcinoma remain unclear given the rarity of this clinicopathology. The current study reports the case of a 65-year-old male who presented with advanced thymic carcinoma with solitary brain and pulmonary metastases, but demonstrated long-term survival following multiple lines of chemotherapy and radiotherapy with palliative intent. Although the solitary brain metastasis was well controlled for several years using whole-brain irradiation, cognitive function gradually declined with cerebral atrophy. Thymic carcinoma is known to show a poor prognosis and aggressive clinical progress, however, it occasionally demonstrates a clinically indolent course. Modalities of treatment should thus be selected prudently to avoid toxicity, in consideration of the possibility of long-term survival. Stereotactic radiation therapy for brain metastases, including cyberknife or γ-knife surgery, appears to represent the optimal local treatment for such patients with unexpectedly longer survival due to indolent thymic carcinoma.

## Introduction

Thymic carcinoma is a rare cancer with a poor prognosis (5-year survival rate, 20–30%; 2-year survival rate, 50%) due to rapid extension in comparison with thymoma (1). Thymic carcinoma was first reported by Shimosato *et al,* and later, was classically distinguished from type C thymoma (2). A total of 13 subtypes have been defined according to the classification of the World Health Organization (WHO) (3). Due to the presence of thymic non-organotypia, no symptomatic paraneoplastic syndrome appears, so patients are typically diagnosed with progressive disease when they initially present with symptoms associated with tumor extension. In terms of prognostic factors, staging and grade of atypia are significant (4,5). Definitive clinical management and treatment have usually been determined with a high level of evidence for common cancers, whereas clinical entities are unavailable for rare cancers, such as thymic carcinoma, due to the lack of large-scale clinical trials. Treatment options are thus selected by the individual physician and appear to be determined according to the prognosis.

The present study reports a rare case of long-term survival for 10 years in a patient with metastatic thymic carcinoma who could be treated using chemotherapy and radiotherapy for local control with palliative intent. Patient provided written informed consent.

## Case report

**Figure 1:**
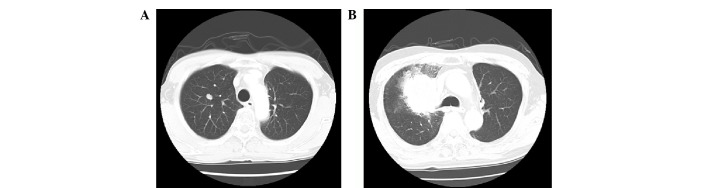
Contrast-enhanced computed tomography of the chest in 2006 revealing pulmonary metastasis in S1 of the right upper lobe (RUL). (A) Enhancement 1×2 cm in size. (B) The pulmonary metastasis in the RUL in the ninth year.

**Figure 2:**
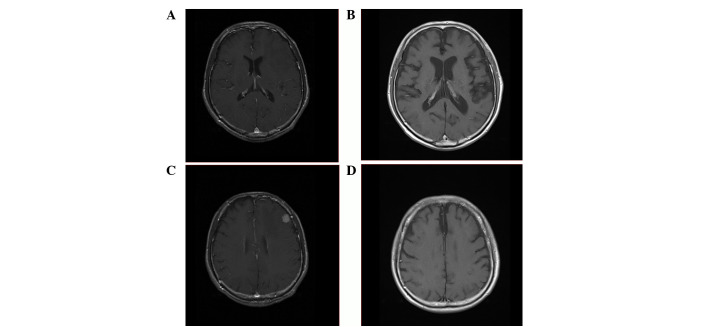
(A) Brain metastasis at presentation prior to whole-brain radiotherapy. (B) No recurrence for brain metastasis or evident brain atrophy was present on magnetic resonance imaging 10 years after the initiation of treatment, however, (C and D) cerebral atrophy was shown to be gradually exacerbated on the images.

**Figure 3:**
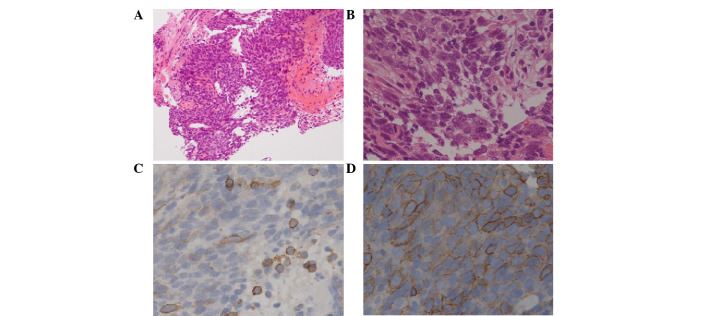
In the right upper lobe (S1), (A and B) the pulmonary metastasis was revealed to be thymic carcinoma with squamous histology [hematoxylin and eosin stain; (A) magnification, ×40, (B) magnification, ×400] and (C) appeared positive for cluster of differentiation 5 (magnification, ×200) and (D) appeared positive for c-kit (magnification, ×400) in the ninth year by immunohistochemical staining.

A 65-year-old male presented to the Tokyo Metropolitan Cancer and Infectious disease Center Komagome Hospital (Tokyo, Japan) with a dry cough and mild hemoptysis. The patient had been treated with chemotherapy since 2002 for advanced thymic squamous cell carcinoma, and showed solitary cerebral and pulmonary metastases in the right lower lobe (RLL) on presentation. At the time of the initial diagnosis, a specimen was acquired from the primary site using computed tomography-guided biopsy. First-line chemotherapy was comprised of cisplatin (80 mg/m^2^ on day one, every 28 days) and irinotecan (60 mg/m^2^ on days one, eight and 15, every 28 days). The patient subsequently underwent irradiation of the primary site and pulmonary metastasis in the RLL with a total dose of 60 Gy over 6 months, followed by whole-brain irradiation (WBI) for the solitary brain metastasis with a dose of 40 Gy two years into therapy (Fig. 1). In terms of the clinical course, cognitive function gradually declined due to WBI, and cerebral atrophy was shown on head imaging (Fig. 2). Pulmonary metastasis in the RUL was histologically confirmed as thymic squamous cell carcinoma following transbronchial biopsy (Fig. 3). Histologically, the tumor was composed of highly atypical epithelioid cells, with nuclear atypia and eosinophilic cytoplasms in the fibrotic stroma (Fig. 3A and B). Immunohistochemically, the tumor cells were positive for CD5 and c-Kit (Fig. 3C and D). The patient was treated with nine lines of chemotherapy and palliative radiotherapy for local control of the brain metastasis and two pulmonary metastases. The patient then underwent second-line chemotherapy comprised of two cycles of carboplatin (area under the curve 6 on day one, every 21 days)/paclitaxel (80 mg/m^2^ on days one, eight and 15, every 21 days). In the fourth year, pulmonary metastasis in the RUL was targeted and irradiated once at a dose of 60 Gy, but recurrence subsequently developed. Following completion of radiation therapy, the patient received six lines of chemotherapy in 4 years, comprised of four cycles of gemcitabine (800 mg/m^2^ on days one and eight, every 21 days) and vinorelbine (25 mg/m^2^ on days one and eight, every 21 days), two cycles of docetaxel (60 mg/m^2^ on day one, every 21 days), 12 cycles of S-1 (80 mg/m^2^ on days one to 14, every 21 days), four cycles of amrubicin (35 mg/m^2^ on days one to three, every 21–28 days), eight cycles of irinotecan (100 mg/m^2^ on days one, eight and 15, every 28 days), three cycles of pemetrexed (500 mg/m^2^ on day one, every 21 days), and lastly, the combination of four cycles of doxorubicin 40 mg/m^2^, cisplatin (50 mg/m^2^ on day one, every 21 days), vincristine (0.6 mg/m^2^ on day three, every 21 days) and cyclophosphamide (700 mg/m^2^ on day four, every 21 days) (ADOC). The patient succumbed to carcinomatous lymphangiosis in the 10th year of treatment following the initiation of first-line chemotherapy.

## Discussion

The patient in the present study presented with thymic carcinoma with distant metastasis at the time of the initial diagnosis (stage IVb according to the Masaoka-Koga staging system) and was a long-term survivor who was treated for 10 years with chemotherapy and radiotherapy for local control with palliative intent. Local irradiation enabled control of the primary site and pulmonary metastasis in the RLL. Although the brain metastasis was also well controlled with WBI, cognitive function declined as a result of this treatment.

Thymic carcinoma is classified as a type C thymoma with strong atypia according to the 1999 WHO classification, on the basis of the classification by Müller-Hermelink *et al* (6). In the 2004 WHO classification, thymic carcinoma was categorized separately from thymoma. Histological subtypes of thymic carcinoma include squamous and lymphoepithelioma-like carcinoma, comprising 60–70% of cases (3). A diagnosis can be reached using immunohistochemical staining for cluster of differentiation (CD)5 and c-kit, with c-kit expression appearing more frequently in thymic carcinoma (75%) than in thymoma (2%) (7). The expression of insulin-like growth factor 1 in thymic epithelial tumors is associated with prognosis in these patients (8). As there is a loss of thymic organo-specific characteristics that induce CD4/CD8 double-positive T cells, as seen in myasthenia gravis and pure red cell aplasia, symptomatic paraneoplastic syndrome does not appear. Patients with thymic carcinoma are thus usually diagnosed with progressive disease subsequent to presenting with symptoms associated with tumor extension.

As first-line chemotherapy for advanced thymic carcinoma, cisplatin and anthracycline-based chemotherapies, such as ADOC (9) and the combination of cisplatin, Adriamycin and cyclophosphamide (10), are applied in the clinical setting based on Einhorn’s protocol for germ cell tumors. Only a prospective phase II trial with carboplatin and paclitaxel for unresectable stages has been performed, indicating the efficacy of platinum-based doublet chemotherapy (11). With regard to second-line chemotherapies, evidence for efficacious regimens has not been presented and almost all reported series have included only small numbers of patients (12). In the present case, ad hoc treatment with singlet chemotherapy or local treatment with radiotherapy were compatible with long-term survival. Each treatment period was not particularly long, but progression-free intervals were modest. In previously reported cases, the prognosis of stage IVb tumors, as classified in the Masaoka-Koga Stating System of thymic carcinoma, was in the range of 19–46 months (13). In the present case, nine lines of chemotherapy appeared to be beneficial for inhibiting disease progression as salvage chemotherapy, although clinical progress is rarely indolent. We previously documented a first-line chemotherapy response rate of 47.5% and a median survival time of 24.5 months. The overall survival rates at 1, 2, and 5 years were recorded as 72.5, 52.5 and 17.5%, respectively (14). In general, thymic carcinoma demonstrates an aggressive clinical course, but ~20% of patients treated with palliative-intent chemotherapy for advanced thymic carcinoma survive for 5 years. Oncologists should be mindful of the fact that a substantial proportion of patients with advanced thymic carcinoma show an indolent clinical process. In the present case, the primary site, pulmonary metastasis at initial diagnosis and solitary brain metastasis were locally controlled using radiotherapy alone at a dose of 40–60 Gy. No evidence of recurrence was demonstrated radiologically at the end stage in this patient. In terms of the clinical process, the thymic carcinoma was indolent and sensitive to treatment. However, cognitive function was adversely affected by whole-brain metastasis, with an unexpected longer survival time. Patients with indolent thymic carcinoma may survival longer, so stereotactic radiotherapy, such as cyberknife or γ-knife surgery, is reasonable to minimize the effects on cognitive function.

In conclusion, thymic carcinoma is known to have a poor prognosis and an aggressive clinical process, however, clinically indolent patients with advanced thymic carcinoma are occasionally encountered. Treatment modalities should thus be prudently selected while considering the possibility of long-term survival, although optimal management for advanced thymic carcinoma has yet to be defined due to the rarity of this pathology. A high level of evidence for the clinical management of rare cancers cannot realistically be determined in large prospective clinical studies, so longer follow-up periods for minimally invasive treatments are warranted. Retrospective multiple-institution registries will also provide useful clues to deciding on the clinical management in such rare cancers.

## References

[1] Eng TY, Fuller CD, Jagirdar J, Bains Y, Thomas CR (2004). Thymic carcinoma: state of the art review. Int J Radiat Oncol Biol Phys.

[2] Shimosato Y, Kameya T, Nagai K, Suemasu K (1977). Squamous cell carcinoma of the thymus. An analysis of eight cases. Am J Surg Pathol.

[3] Travis WD, Brambilla E, Müller-Hermelink HK, Harris CC (2004). World health organization classification of tumors. Pathology and Genetics of Tumors of the Lung, Pleura, Thymus and Heart.

[4] Okumura M, Ohta M, Tateyama H (2002). The world health organization histologic classification system reflects the oncologic behavior of thymoma: a clinical study of 273 patients. Cancer.

[5] Quintanilla-Martinez L, Wilkins EW, Choi N, Efird J, Hug E, Harris NL (1994). Thymoma. Histologic subclassification is an independent prognostic factor. Cancer.

[6] Müller-Hermelink HK, Marino M, Palestro G (1986). Pathology of thymic epithelial tumors. Curr Top Pathol.

[7] Petrini I, Zucali PA, Lee HS (2010). Expression and mutational status of c-kit in thymic epithelial tumors. J Thorac Oncol.

[8] Mimae T, Tsuta K, Kondo T (2012). Protein expression and gene copy number changes of receptor tyrosine kinase in thymomas and thymic carcinomas. Ann Oncol.

[9] Fornasiero A, Daniele O, Ghiotto C (1991). Chemotherapy for invasive thymoma. A 13-year experience. Cancer.

[10] Loehrer PJ, Kim K, Aisner SC, The Eastern Cooperative Oncology Group, Southwest Oncology Group, and Southeastern Cancer Study Group (1994). Cisplatin plus doxorubicin plus cyclophosphamide in metastatic or recurrent thymoma: final results of an intergroup trial. J Clin Oncol.

[11] Lemma GL, Lee JW, Aisner SC (2011). Phase II study of carboplatin and paclitaxel in advanced thymoma and thymic carcinoma. J Clin Oncol.

[12] National Comprehensive Cancer Network Thymomas and thymic carcinomas (version 1. 2014).

[13] Girard N (2013). Thymic epithelial tumours: From basic principles to individualised treatment strategies. Eur Respir Rev.

[14] Okuma Y, Hosomi Y, Takagi Y (2013). Clinical outcomes with chemotherapy for advanced thymic carcinoma. Lung Cancer.

